# A support vector machine based test for incongruence between sets of trees in tree space

**DOI:** 10.1186/1471-2105-13-210

**Published:** 2012-08-21

**Authors:** David C Haws, Peter Huggins, Eric M O’Neill, David W Weisrock, Ruriko Yoshida

**Affiliations:** 1 Department of Statistics, University of Kentucky, 725 Rose Street, Lexington, KY 40536-0082, USA; 2 Department of Biology, University of Kentucky, 101 TH Morgan Building, Lexington, KY 40506, USA; 3 Robotics Institute, Carnegie Mellon University, 5000 Forbes Ave, Pittsburgh, PA 15213, USA

## Abstract

**Background:**

The increased use of multi-locus data sets for phylogenetic reconstruction has increased the need to determine whether a set of gene trees significantly deviate from the phylogenetic patterns of other genes. Such unusual gene trees may have been influenced by other evolutionary processes such as selection, gene duplication, or horizontal gene transfer.

**Results:**

Motivated by this problem we propose a nonparametric goodness-of-fit test for two empirical distributions of gene trees, and we developed the software GeneOut to estimate a p-value for the test. Our approach maps trees into a multi-dimensional vector space and then applies support vector machines (SVMs) to measure the separation between two sets of pre-defined trees. We use a permutation test to assess the significance of the SVM separation. To demonstrate the performance of GeneOut, we applied it to the comparison of gene trees simulated within different species trees across a range of species tree depths. Applied directly to sets of simulated gene trees with large sample sizes, GeneOut was able to detect very small differences between two set of gene trees generated under different species trees. Our statistical test can also include tree reconstruction into its test framework through a variety of phylogenetic optimality criteria. When applied to DNA sequence data simulated from different sets of gene trees, results in the form of receiver operating characteristic (ROC) curves indicated that GeneOut performed well in the detection of differences between sets of trees with different distributions in a multi-dimensional space. Furthermore, it controlled false positive and false negative rates very well, indicating a high degree of accuracy.

**Conclusions:**

The non-parametric nature of our statistical test provides fast and efficient analyses, and makes it an applicable test for any scenario where evolutionary or other factors can lead to trees with different multi-dimensional distributions. The software GeneOut is freely available under the GNU public license.

## Background

Systematists often wish to compare gene trees, or sets of trees, to each other in a statistical framework and ask whether or not they are significantly different. These efforts have been more traditionally applied to the evaluation of competing phylogenetic hypotheses [1,2]. For example, in the analysis of a single data set, a tree reconstructed in an unconstrained search can be compared to a tree reconstructed under a topological constraint to calculate the difference in tree scores. When compared to the distribution of tree-score differences calculated in a series of simulated data sets, the systematist can determine if their data reject alternative phylogenetic hypotheses [3]. More recently, with the growth of multi-locus phylogenetic data sets, this need has also grown to compare trees generated from different genomic regions, spurring the development of a number of different methods for assessing concordance or discordance among trees across genes [4]. In addition, comparisons need not be restricted to trees generated from analyses of separate data sets. For example, Markov chain monte carlo (MCMC) phylogenetic analyses require a user to determine when independently-run MCMC analyses of the same data set have converged on the same posterior distribution of trees. Often this is assessed through the comparison of simple summary statistics such as the distribution of log likelihood scores or through visualization methods that permit comparisons of the tree topology across independent runs [5].

Overall, this is not meant to be an exhaustive list of situations where trees, or sets of trees, need to be compared with each other, but it highlights a general need in phylogenetics for tools to assess congruence, particularly from a statistical perspective. A non-parametric test is a preferable tool to use for these purposes in light of the growing availability of phylogenomic data sets because of the simplicity in its implementation and efficiency in providing results.

Projecting and visualizing trees in a multi-dimensional framework provides a useful mechanism for comparing large numbers of phylogenetic trees [6,7]. For example, pairwise distances between trees can be calculated using a variety of metrics (e.g., Robinson-Foulds distances) and these matrices can be analyzed using multi-dimensional scaling techniques to plot tree-to-tree distances in ordination space [6]. Another example is the software AWTY for a visual comparison of the posterior distributions from two runs of Bayesian tree construction analysis [5]. These methods can be informative in highlighting differences in pre-defined sets of trees (e.g., [8]). However, few actual statistical tests are available for distinguishing between pre-defined sets of trees that have significantly different multi-dimensional distributions.

Here we propose a non-parametric test combined with a permutation test and the use of support vector machines (SVMs) as a quantitative tool of a statistical test to determine if sets of vectorized gene trees have significantly different multi-dimensional distributions. SVMs can be applied to any two collections of trees which may or may not have been sampled from the same underlying distribution (e.g., reconstructed gene trees for host and parasite species), or two posterior sets of trees independently generated from Bayesian analysis of a single dataset. From a practical perspective, a major reason for the popularity of SVMs in machine learning is their efficiency and accuracy at classifying data in a high dimensional vector space (see [9] for a recent review of SVMs along with biological applications).

**Figure 1:**
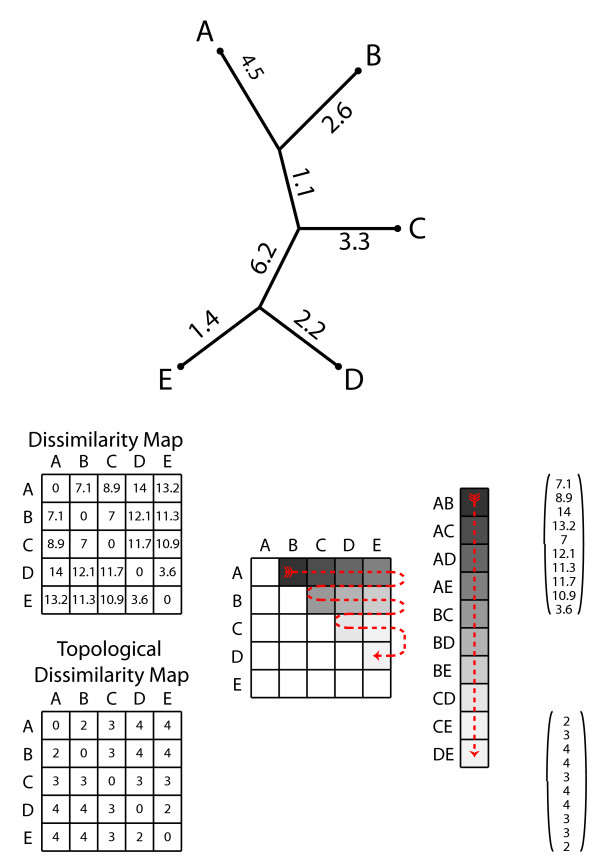
**Schematic of how trees are converted to vectors.** Numbers on branches in the unrooted tree are branch lengths. In this example, the tree is first converted to either a branch length-based dissimilarity map (matrix of distances between tips) or topological dissimilarity maps (matrix of number of edges between tips). Moving from left to right across rows in one half of a matrix, values are placed into a single column to yield a vector of distances between tips in the tree.

In our approach, trees can be incorporated into a statistical framework by converting them into a numerical vector format based on a distance matrix or map, see Figure 1. These vectorized trees can then be analyzed as points in a multi-dimensional space where the distance between trees increases as they become more dissimilar [6,10,11]. While these methods are effective in the evaluation of large numbers of trees, they have primarily been used in the qualitative visualization of tree space [6,12] or statistical applications that test for incongruence simply between two trees [7,13].

**Figure 2:**
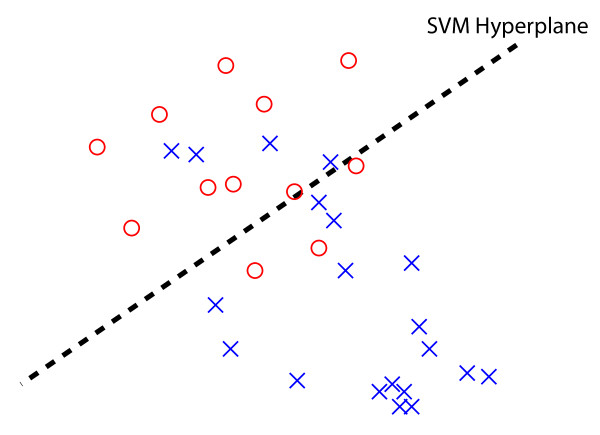
**A two dimensional example of a support vector machine (SVM) training to find the best hyperplane (dashed line) separating two pre-defined groups of points (labeled ’x’ and ’o’).** In this example, the hyperplane correctly classifies 9 of the 12 o’s and 16 of the 20 x’s, and thus the data has a separation percentage of (9 + 16)/32 = 0.78125 , or 78.125*%*.

SVMs are supervised learning algorithms that can be used to compute the separation between two sets of points, or point-clouds, in a multi-dimensional space [14]. Given two sets of points *X*_ + _ and *X*_−_ in high dimensional space, an SVM finds a hyperplane *H* that maximizes linear separation between *X*_ + _ and *X*_−_ (see Figure 2) while attempting to avoid overfitting. The hyperplane splits multidimensional space into two half-spaces *H*^ + ^ and *H*^−^ . The separation percentage *δ* is half the percentage of points of *X*_ + _in *H*^ + ^ , plus half the percentage of points of *X*_−_in *H*^−^ . For data sets *X*_ + _ and *X*_−_ which are not entirely separable, the separation percentage produced by the SVM hyperplane is a quantitative and intuitive measure of separation. Overall, the classification of data with SVMs is a two-step process. In the first step (i.e. training), the SVM algorithm uses a set of pre-classified examples each belonging to one of two categories to learn a hyperplane that maximizes an objective that balances between separating the two categories while avoiding overfitting. In the second step (i.e. testing), new examples are mapped into the same space and predicted to belong to a category based on which side of the established hyperplane they fall.

To implement SVMs in the statistical testing of tree distributions, we developed a permutation test, augmented by bootstrapping for application to DNA sequence alignments, that assesses the significance of SVM separation percentages between two predefined sets of vectorized trees in multidimensional space. We emphasize that the SVM separation alone is not an indication that the two sets of trees are incongruent. That is, the SVM separation percentage is only relevant when compared to all possible SVM separation percentages when permuting the data. For example, suppose 100 gene trees were sampled under the coalescent. Most likely the trees will not be identical but the SVM separation percentages will be indistinguishable for all possible test with 1 tree in one set and the other 99 trees in the other test, implying that no single tree will appear as an outlier. Also, we note that the SVM separation percentages may be above 50% and this does not present a problem as all other SVM separation percentages when permuting the data will be similar.

To demonstrate the utility of our statistical test in discriminating between different sets of trees, we apply it in a simulation study that compares gene trees sampled from two different eight-taxa species trees. By varying the total depth of the species trees, this framework serves as a general proxy for generating sets of trees with varying levels of overlap in multidimensional space. In addition to exploring the sensitivity of our statistical test in detecting differences among gene tree distributions, we also explore its performance using different mapping techniques (dissimilarity maps vs. topological dissimilarity maps) and tree reconstruction methods (Bayesian, Maximum Likelihood, and Neighbor Joining). Finally, we assess the scalability of our statistical test to trees with larger numbers of taxa.

## Methods

### Representing trees as vectors

To apply SVMs, we represent gene trees as vectors as follows. Given a tree *T* with *n* taxa, the dissimilarity map of *T* is the *n*×*n* matrix whose (*i, j*)th entry is the sum of the branch lengths between taxa *i* and *j*[15]. Similarly, the topological dissimilarity map of *T* is the *n*×*n*matrix whose (*ij*) th entry is the number of branches between taxa *i* and *j*. This is also called the vector of branching numbers (see page 531 in [16]) and the vector of path differences [17]. Note that the topological dissimilarity map is the dissimilarity map when each branch length of *T* is set to 1. We represent a dissimilarity map by a vector by lexicographically listing the upper diagonal entries of the matrix: [(1,2),(1,3),…,(1,*n*),(2,3),(2,4),…,(2,*n*),(3,4),…,(*n* − 1,*n*)] . For a tree with *n* taxa, the resulting vector is of length n2=n(n−1)/2 . Figure 1 provides a visual depiction of this process. Both the dissimilarity map and topological dissimilarity map have the desirable properties that they can be computed quickly, and represent trees by vectors of relatively low dimension ( n2 for trees with *n* taxa).

### Testing for incongruence between sets of reconstructed gene trees using SVM

We present a goodness-of-fit test, which takes two sets of sequence alignments as input and tests the null hypothesis that the underlying distributions of phylogenetic trees are the same. We require some terminology in order to state our formal hypothesis. Suppose gene trees have been mapped into m-dimensional real space ( $R^{m}$) where *m* = *n*(*n* − 1)/2 and *n* is the number of leafs in the trees. Given two distributions *p, q* over trees, we define the separation percentage *δ* to be $\underset{H^{+}}{\max}\frac{1}{2}(p(H^{+})\;+1\;-q(H^{+}))$, where the max is taken over all half-spaces *H*^ + ^ . Here the notation *p*(*H*^ + ^) denotes the total probability (under *p*) of all trees in *H*^ + ^ , and similarly for *q*(*H*^ + ^) . That is, any half-space *H*^ + ^ will contain a subset (or all) of all possible vectorized trees in *R*^*m*^ . Then *p*(*H*^ + ^) is the total probability of the trees contained in the half-space *H*^ + ^, i.e. $p(H^{+}):=\int_{H^{+}}\mathrm{dp}$ . Similarly for *q*(*H*^ + ^) .

Our statistical hypotheses is

$$\;\begin{array}{c} H_{0}:\text{Two sets of trees are drawn from the same}\;\\ \text{distribution.}\;\;\;\;\;\\ H_{1}:\text{Two sets of trees are not drawn from the same}\\ \text{distribution.}\;\;\;\;\; \end{array}$$

In a model where trees are generated according to a distribution *p*, and then DNA alignments are generated on each tree, trees reconstructed from alignments are not direct samples from the original distribution *p*. As an example, for gene trees generated by a coalescent model, reconstructed gene trees are not merely samples from the coalescent, but also are influenced by the observed sequence data and choices of gene tree inference. We do not know the null distribution of the separation percentages; hence, we develop methods to estimate the null distribution. Again we emphasize that in practice we often observe SVM separation percentages above 50% but we can only reject the null hypothesis when we evaluate this separation percentage in light of the null distribution (estimated by a permutation test).

**Figure 3:**
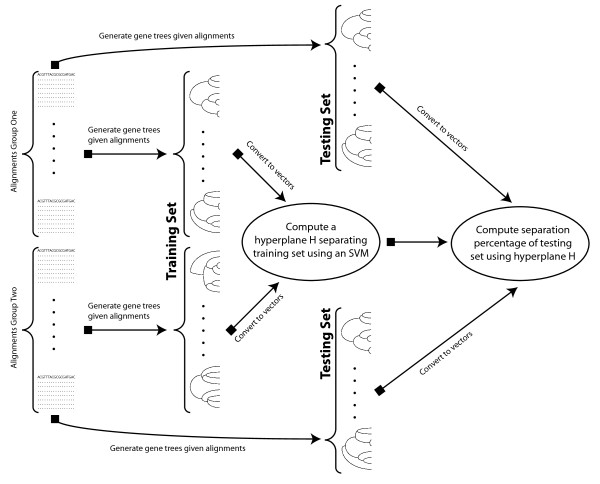
**A flowchart describing how our statistical test calculates the separation percentage of the evolutionary histories of two sets of DNA sequence alignments, A and B.** First, gene trees, labeled “training set”, are inferred from alignments A and B. The training set gene trees are vectorized and an SVM is trained to find a hyperplane separating the vectorized gene trees. Next, a new set of gene trees, labeled “testing set”, are inferred from alignments A and B. The testing set gene trees are vectorized and the hyperplane previously computed by the SVM is used to calculate a separation percentage.

Our statistical test includes a novel non-parametric statistical procedure that estimates a p-value for the statistical hypotheses described above, from input DNA sequences. At the core of our statistical test is the sub-process of using an SVM to compute a separation percentage between vectorized gene trees inferred from two sets of DNA sequences. This sub-process is outlined in Figure 3 and is described as follows. Our test takes two sets of DNA sequence alignments *A* = {*A*_1_,…,*A*_*m*__1_} and *B* = {*B*_1_,…,*B*_*m*__2_} as input, shown in the left of Figure 3. From each set of alignments, *A* and *B*, two sets of gene trees, *T*_*A*_ and *T*_*B*_ respectively, are inferred. These are labeled “training set” in Figure 3. The inferred trees *T*_*A*_ and *T*_*B*_ (training set) are vectorized and an SVM is used to compute a separating hyperplane, as depicted in the center oval of Figure 3. Again, from each set of alignments *A* and *B* two different sets of gene trees $T_{A}^{\prime }$ and $T_{B}^{\prime }$ are inferred. These are labeled “testing set” in Figure 3. The inferred trees $T_{A}^{\prime }$ and $T_{B}^{\prime }$ (testing set) are vectorized and the previously computed hyperplane is used to calculate the separation percentage between the vectorization of $T_{A}^{\prime }$ and $T_{B}^{\prime }$. This final step is shown in the right oval of Figure 3. Finally, the separation percentage test statistic $\hat{\delta }$ is recorded.

In order to estimate the null distribution *δ*, this sub-process is repeated multiple times with hypothetical data sets *A*^∗^ and *B*^∗^ generated by a permutation procedure as follows. First alignment labels are permuted to create hypothetical sets of alignments *A*^∗^,*B*^∗^ . Then each alignment in *A*^∗^is replaced by a bootstrap replicate with the same number of columns as the corresponding alignment in *A* (and similarly for *B*^∗^). See the appendix for pseudo-code of the GeneOut procedure. The set of alignment sizes in *A*^∗^,*B*^∗^ is identical to *A, B*, but each alignment column in *A*^∗^and *B*^∗^follows the same marginal empirical distribution derived from *A* union *B*.

In the GeneOut procedure, each time trees are inferred from alignments, the user can specify that multiple trees are inferred from each alignment. For a single-tree reconstruction method like NJ or ML, this means the user can specify that several bootstrap trees are reconstructed for each alignment. For a Bayesian reconstruction method, the user can specify that multiple samples are taken for each posterior distribution of trees. Reconstructing multiple trees for each alignment allows the SVM separation to take into account uncertainty in tree reconstruction. In the GeneOut procedure, we allow more than one reconstructed tree per alignment, via a parameter *M* that specifies how many total trees should be sampled for each set of alignments. See the pseudo-code for details. Note that in the above description, the choice of tree reconstruction method is not specified; any statistically consistent tree reconstruction method can be used.

Our use of the SVM separation percentage is motivated by the observation that systematic differences between sets of trees may manifest as a separating direction in feature space (e.g. if tree space is defined by using splits as features, then a separating direction indicates which splits tend to occur in one set of trees and not the other). The SVM tries to find a maximal separating direction by deep analysis of the data, without making Gaussian assumptions like Fisher’s linear discriminant. Furthermore, for two sets of points with high variance and a small but reliable separation (e.g. two parallel discs with only a small separation between), the separation statistic gives a more representative indicator of how likely the two point sets come from different distributions, versus distance-only statistics such as comparing within group to between group variance [18]. The power of the SVM separation percentage is also naturally robust to many unusual configurations of points (e.g. generated by mixture models) – the only requirement for statistical power is that a separating hyperplane can be found which causes some appreciable imbalance between the two point sets on either side of the plane.

## Results and discussion

To obtain simulated trees with different distributions, we used coalescent-modeled gene trees simulated within different species tree histories. We first simulated pairs of species trees (*S*_1_ , *S*_2_ ) with *n* = 8 lineages using a pure-birth (Yule) model [19], with a fixed effective population size (*N*_*e*_) of 100,000 haploid individuals, and various tree depths ranging from 0.1*N*_*e*_ to 10*N*_*e*_ . We then simulated sets of 10,000 gene trees (denoted *T*_1_ and *T*_2_) under the respective species tree histories using a neutral coalescent model. In addition, for the purpose of assessing false positive rates (see below), we generated an additional set of 10,000 gene trees (*T*_3_ ) within *S*_2_ using the same process and model parameters used for *T*_2_. These simulation conditions were chosen to represent a broad range of coalescent gene trees within each species tree. For example, at low species tree depth we expect considerable variation among gene trees within a species tree, causing overlap in multidimensional space among gene trees from different species trees. All species tree and gene tree simulations were performed in Mesquite v2.72 [20].

To independently assess the variation between sets of gene trees simulated under different species tree at different species tree depths, we used principal component analysis (PCA) and Fisher’s linear discriminant (FLD) [21]. Specifically FLD projects *T*_1_ and *T*_2_ onto a line which maximizes the distance between the means of *T*_1_ and *T*_2_ while minimizing the variance within *T*_1_ and *T*_2_ . Larger values of FLD indicate greater separation between different sets of gene trees. Because these data are in high dimensions we used PCA to reduce the dimensionality of the data. To visualize separation between *T*_1_ and *T*_2_ , we graphed the first two principal components for each gene tree at each species tree depth. Both FLD and PCA were applied to gene trees vectorized using the dissimilarity map.

To simulate DNA sequence data, we used the simulated gene trees described above. For each gene tree we simulated sequences of 1,000 nucleotides under a Hasegawa-Kishio-Yano (HKY)+*Γ* model [22,23] with a transition-transversion ratio of 3.0 , and a discrete *Γ* distribution with four rate categories and a shape parameter of 0.8 . In each data set we assigned the stationary probability distribution *Π* :=(*Π*_*A*_*Π*_*C*_*Π*_*G*_*Π*_*T*_) = (0.3,0.2,0.2,0.3) : and maintained an *AT:GC* ratio equal to 3:2 throughout the gene tree. The coalescent gene trees had branch lengths in terms of coalescent units; therefore, a branch-length scaling factor of 3 × 10^−8^ was used. These parameters were similar to those used in other recent studies of gene tree evolution within species trees [24], and resulted in pairwise DNA sequence divergences similar to the sequence divergences in Table 1 of [24]. All DNA sequences were generated using Mesquite v2.72.

**Table 1:** Average and minimum pairwise uncorrected percent sequence divergences calculated from simulated DNA sequence data

**Species tree depth**	**Average pairwise**	**Average minimum**
**(in *N*_*e*_ generations)**	**sequence divergence**	**sequence divergence**
0.1	0.9371(0.3631)	0.08(0.0356)
0.2	1.0410(0.3589)	0.1(0.0570)
0.3	1.0910(0.3832)	0.1(0.06378)
0.4	1.2010(0.3763)	0.1(0.0790)
0.6	1.0510(0.3645)	0.14(0.0948)
0.8	1.2590(0.3757)	0.18(0.1066)
1.0	1.3630(0.3860)	0.24(0.1219)
2.0	1.9040(0.4014)	0.42(0.2092)
4.0	2.6340(0.5113)	0.62(0.2092)
6.0	3.437(0.5556)	0.82(0.4014)
8.0	4.409(0.5312)	0.54(0.3151)
8.5	3.787(0.6200)	0.7(0.3406)
9.0	4.281(0.7800)	0.62(0.2801)
9.5	4.311(0.5041)	0.52(0.3124)
10.0	4.426(0.5165)	0.8(0.4001)

Divergences were calculated using all 3000 simulated data sets for a species tree depth (1000 from the first replicate species tree and 2000 from the second replicate species tree). Standard deviations are given in parenthesis. All species trees were simulated using an *N*_*e*_ of 100,000.

For gene tree reconstruction we used NJ under the Felsenstein 84 (F84) model [25] in the software package PHYLIP v3.6 [26], and ML under the HKY + *Γ* model using the software PhyML[27]. We also used MrBayes v3.1.2 [28] with HKY + *Γ* model to obtain posterior samples. Convergence statistics for the MCMC sampling were within the guidelines suggested in the MrBayes v3.1.2. manual. See Additional file 1 for more details about how MrBayes was run.

### Simulation study using simulated gene trees

In reality, we estimate phylogenetic trees from observed data so that these trees are subject to uncertainty at some level. Thus, in order to determine our statistical tests’ inherent ability to detect separation of the underlying distribution of trees, we first performed a series of experiments where we assume all trees are the true trees. To asses the true positive and false negative rates of our statistical test we conducted our statistical hypothesis test with two samples of gene trees generated under the distributions of different species trees. Similarly, to asses the true negative and false positive rates we conducted our statistical hypothesis test with two samples of gene trees generated under the distributions of the same species tree.

For the first type of tests (assessing true positive and false negative rates) we ran our statistical test using, as input, 10,000 gene trees *T*_1_ and 10,000 gene trees *T*_2_ . We calculated a separation percentage by training and testing an SVM with 168 and 336 (respectively) gene trees sampled from *T*_1_ and *T*_2_ . That is, we sampled 168 gene trees from *T*_1_ , and 168 gene trees from *T*_2_ , and trained an SVM. Next, we sampled 336 gene trees from *T*_1_, and 336 gene trees from *T*_2_, and we used the previously trained SVM to compute the separation percentage. We calculated the separation percentage 100 times and took its average. We approximated the null distribution by repeating the following 100 times: we trained and tested an SVM with 168 and 336 gene trees sampled just from *T*_2_ . We estimated a p-value using the separation percentage and the null distribution approximation. We performed this statistical test for all fifteen species tree depths and using either the dissimilarity or topological dissimilarity map vectors.

For the second type of tests (assessing true negative and false positive rates) we ran our statistical test using, as input, 10,000 gene trees *T*_2_ and 10,000 gene trees *T*_3_ . We calculated a separation percentage by training and testing an SVM with 168 and 336 (respectively) gene trees sampled from *T*_2_ and *T*_3_ . We calculated the separation percentage 100 times and we took its average. We approximated the null distribution by repeating the following 100 times: we trained and tested an SVM with 168 and 336 gene trees sampled just from *T*_3_ . We estimated a p-value using the separation percentage and the null distribution approximation. We performed this test for all fifteen species tree depths and using either the dissimilarity or topological dissimilarity map vectors.

### Simulation study using simulated DNA sequences

We explored a range of options when testing our statistical test in order to assess the effects of balanced vs. unbalanced sets, species tree depth, tree reconstruction method, and tree vectorization method. To test our statistical tests’ ability to detect separation when the underlying tree distributions were not the same, we performed statistical tests with alignments generated from gene trees within different species trees. To assess false positive error, we also performed tests where the alignments were generated from gene trees within the same species tree. We fixed four conditions for all tests: We computed the separation percentage 100 times and we took its average, we repeated the permutation sub-process 100 times in order to estimate the null distribution, and we used the SVM training and testing phases with samples sizes of 168 and 336, respectively. Our statistical test takes, as input, two sets of DNA sequence alignments *A* and *B* (described above). We described our experiments in terms of *T*_1_,*T*_2_,*T*_3_ defined above. The experiments we performed fall into three categories determined by the number of alignments in *A* and the number of alignments in *B*: 1 vs. 10, 1 vs. 50 and 10 vs. 10, each with two sub-categories. The sub-categories correspond to tests where the species trees are different and the species trees are the same. The three categories are summarized as follows.

**1 vs. 10**: We selected the first ten alignments generated from y and the first ten alignments generated from *T*_2_ . We denoted the two sets of ten alignments *L* and *R*. For each alignment *A* of *L* we ran GeneOut with input *A* and *R*, resulting in ten tests. We performed these ten tests for all fifteen species tree depths, using Neighbor Joining (NJ), Maximum Likelihood (ML), and Bayesian Inference (BI) tree reconstruction methods, and using both dissimilarity and topological dissimilarity maps.

We selected the first eleven alignments generated from *T*_2_. We called the set of eleven alignments *R*, and for an alignment *A* in *R* we define *R*−*A* as the set of all alignments in *R* except *A*. For each alignment *A* of *R* we ran GeneOut with input *A* and *R*−*A* , resulting in ten tests. Tests were performed as described in the preceding paragraph.

**1 vs. 50**: We selected the first 50 alignments generated from *T*_1_ and the first 50 alignments generated from *T*_2_. We denoted the two sets of fifty alignments *L* and *R*. For every alignment *A* in *L* we ran GeneOut with input *A* and *R*, resulting in 50 tests. We performed these 50 tests using the NJ tree reconstruction method for all fifteen species tree depths using both dissimilarity and topological dissimilarity maps.

We selected the first 51 alignments generated from *T*_2_ and called the set of alignments *R*. For every alignment *A* in *R* we ran GeneOut with input *A* and *R*−*A* , resulting in 50 tests. Tests were performed as described in the preceding paragraph.

**10 vs. 10**: We selected the first 100 alignments generated from *T*_1_ and the first 100 alignments generated from *T*_2_ . We denoted the two sets of 100 alignments *L* and *R*. Let *L* = *L*_1_,…,*L*_10_ and *R* = *R*_1_,…,*R*_10_ where *L*_*i*_ and *R*_*i*_ are the *i*th set of ten alignments of *R* and *L*, respectively. We selected every pair (*L*_*i*_,*R*_*i*_) of two sets of ten alignments from *R* and *L* and we ran GeneOut with input *L*_*i*_ and *R*_*i*_ , resulting in 10 tests. We performed these ten tests using the NJ tree reconstruction method and performed them for all fifteen species tree depths, using both the dissimilarity and the topological dissimilarity maps. Similarly, we repeated the above experiments with the exception that we selected the first 100 alignments generated from *T*_2_ and the first 100 alignments generated from *T*_3_.

### ROC Curves and False positive plots

To assess the overall accuracy of our statistical test, we used receiver operating characteristic (ROC) [29] curves. A ROC curve is a graphical representation of the true positive rate vs. false positive rate of a binary classifier as a classifier boundary is varied. ROC analysis therefore provides a tool to evaluate a method’s ability to accurately classify data. In our simulation study, the binary classifier was the GeneOut procedure and *α* -level was the classifier boundary. A data set is classified according to whether or not the null hypothesis is rejected (i.e. *p*-value is less than a given *α*-level). A true positive means that GeneOut detects significant separation between two sets of trees when the distributions on trees are not equal, and a false positive means that there is a significant separation when the distributions on trees are equal. To generate each data point on a ROC curve, we first fixed an *α*-level. We then computed the true positive and false positive rates from all the data for the fixed *α* -level. In order to generate the entire ROC curve, we varied the *α*-level from 0 to 1. The diagonal of a ROC graph represents random classification of the data (i.e. true positive rate = false positive rate). Perfect classification (i.e. 100% true positives and 0% false positives) results in a curve that passes through (*x* = 0,*y* = 1 ). Therefore the closer the ROC curve is to the upper left corner, the higher the overall accuracy of the test [30].

We also calculated the area under the curve (AUC) for each ROC curve to provide a summary statistic of classification accuracy. In general terms, the AUC is the probability that a binary classifier will rank a randomly chosen positive example higher than a randomly chosen negative example; therefore the AUC is equivalent to a Wilcoxon signed-rank test. In our simulation study, the classifier was the GeneOut’s procedure, the rank was determined by the p-value, a positive example was a set of gene trees simulated under two different species tree distributions, and a negative example was a set of gene trees simulated under the same species tree distribution. The AUC for a 1:1 diagonal ROC curve (i.e. random classification) is 0.5 , whereas the AUC for a perfect classifier is 1.0 . We compared ROC curves and AUCs across different tree reconstruction methods, sample sizes and tree vectorization methods.

To assess how our statistical test controls false positive rates, we created graphical representations of the false positive rates vs. *α* -levels (levels of significance). Note, *α*is the probability of making a false positive error (rejecting the null hypothesis when the null hypothesis is true). Thus an *α* -level (level of significance) is preset to be the upper bound of the probability of making a false positive error. Therefore in these graphs, the diagonal line (*y* = *x* ) means that a statistical test has the *α*-level as its false positive rates (which is a maximum allowance of false positive rates). If the test has 0% false positive rate (i.e., the probability of rejecting the null hypothesis when the null hypothesis is true is 0), then the curve is x-axis (the line *y* = 0 ). Therefore, if a curve is under the diagonal line (*y* = *x* ) then the test controls false positive rates below the *α*-level. Also the closer the curve is to the lower right corner the lower the false positive rate of the test is. We compared these curves across different tree reconstruction methods and different tree distances.

We computed all empirical plots for false positive rates vs. *α*-levels, ROC curves, and AUC calculations using R[31]. We drew empirical ROC curves by connecting consecutive pairs of plotted points using a “lower staircase”. In other words, if a point (*a, b*) in the plot was lower-left of a point (*c, d*), then we drew segments from (*a, b*) to (*c, b*) and from (*c, b*) to (*c, d*). This gives the most conservative estimate of a ROC curve passing through the points. Similarly for AUC calculations, we calculated the area under the “lower staircase” curves. We did this in an effort to avoid overestimating AUCs.

As described below, NJ reconstruction exhibited competitive performance with ML and BI reconstruction methods in empirical ROC curves and AUCs, and also controlled false positive rates at the desired *α* -level for all choices of *α*-levels. NJ is also computationally fast compared to ML and BI methods. Thus, in order to compare the performance of our statistical test with topological dissimilarity maps and dissimilarity maps, we restricted our simulation study to NJ tree reconstruction for simulation scenarios of 10 vs. 10 and 1 vs. 50 trees.

### Data sets with large numbers of taxa

To evaluate the scalability of our methods for larger numbers of taxa, we tested three larger simulated data sets, with 30, 50, and 75 taxa. We ran GeneOut for each number of taxa, testing 10 alignments from each species tree. The data sets were generated using a framework similar to the 8 taxa data sets, with a fixed (*N*_*e*_) of 100,000 and a tree depth of 100*N*_*e*_ . Within each species tree, we simulated 10 gene trees along with simulated DNA sequence data (again using a process similar to the 8 taxa data), using scaling factors of 3 × *e*^−9^, 3 × *e*^−10^ , 3 × *e*^−10^ for the 30, 50, and 75 taxa data sets, respectively. Because this particular exercise was performed primarily to evaluate the computational time required to scale to larger numbers of taxa, species tree depths were chosen to create “tight” distributions of gene trees with low discordance. For tree reconstruction we used NJ and we vectorized gene trees using the dissimilarity map. We used training and testing set sizes of 100 and 200 and also 200 and 400.

## Simulation results

### Trees in space

The first two principal components of the PCA indicated that, at all species tree depths there was substantial variation in the spread of vectorized gene trees generated under each species tree, and that the amount of overlap between sets of vectorized gene trees, simulated under different species trees, decreased as species tree depth increases (Additional file 1: Figure S1.). This overall pattern was confirmed by the FLD analyses. FLD values for sets of vectorized gene trees, simulated under different species trees, were larger when species tree depth was greater (Additional file 1: Figure S2.). However, FLD values for sets of vectorized gene trees, simulated under the same species trees did not change across species tree depths (Additional file 1: Figure S2.). At the species tree depth of 0.4*N*_*e*_ and lower we observed that between-species tree FLD was less than 0.3106 , indicating very little separation of the gene trees. Thus, our statistical test applied to gene trees generated from species trees with species depths of 0.4*N*_*e*_ and lower were omitted when constructing ROC curves and curves for false positive rates vs. *α*-levels.

### Simulation study using simulated gene trees

**Figure 4:**
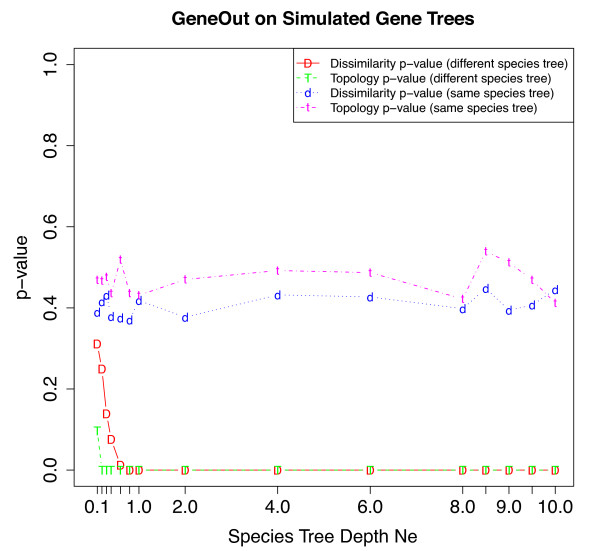
**Our statistical test directly applied to sets of simulated gene trees.** Our statistical test was applied to two sets of 10,000 gene trees, using the dissimilarity and topological dissimilarity maps, and across the fifteen species tree depths. In the first test shown in a line with “D” and in a line with “T”, the two sets of gene trees were generated under different species trees. In the second test shown in a line with “d” and a line with “t”, the two sets of gene trees were generated under the same species tree.

The application of GeneOut directly to simulated gene trees from different species trees resulted in rejection of the null hypothesis (*p* < 0.05 ) at a wide range of species tree depths (Figure 4). When trees were vectorized using topological dissimilarity maps the null hypothesis was rejected for all trees with *N*_*e*_ ≥ 0.1 . However, when trees were vectorized using dissimilarity maps the null hypothesis was rejected for all trees with *N*_*e*_ ≥ 0.6 . Furthermore, when gene trees were generated under the same species tree as input for GeneOut, the null hypothesis was not rejected at any species tree depth and estimated p-values were greater than 0.36 . The dissimilarity maps and topological dissimilarity maps produced similar results.

### Simulation study using simulated DNA sequences

**Figure 5:**
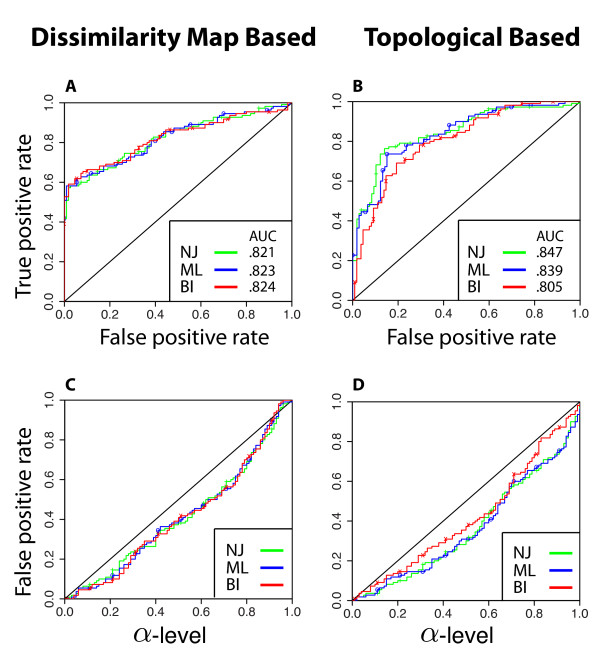
**Comparison of our statistical test performance for three choices of tree reconstruction methods: NJ (red/crosses), ML (blue/circles), and BI (red/X’s).** Trees were reconstructed using PHYLIP, MrBayes and PhyML. **A** and **B** show comparisons of ROC curves on simulated data. See the section *ROC Curves and False positive plots* for a description of the ROC curve. **C** and **D** show comparison of curves on false positive rates (*Y * axis) vs. *α*levels (*X* axis). Panels **A** and **C** are for dissimilarity map-based tree space; panels **B** and **D** are for topological dissimilarity map. In **C** and **D**, the *Y * axis gives the p-values which are less than the *α*level (*X* axis).

In an initial application of our statistical test, using an alignment sampling strategy of 1 vs. 10, all three tree reconstruction methods produced ROC curves that were well above the diagonal and empirical AUCs derived from these curves were all greater than 0.805 (Figure 5). Both of these results indicated a high degree of accuracy in the use of our statistical test to statistically differentiate between different distributions of gene trees. When a dissimilarity map was used to vectorize trees, there was very little difference in performance among NJ, ML, and BI methods (Figure 5A). However, when topological dissimilarity maps were used, NJ exhibited a competitive performance based on an empirical AUC (0.847) compared to ML and BI reconstruction methods (0.839 and 0.805 , respectively) (Figure 5B). In other words, all three reconstruction methods performed similarly well. Furthermore, all three reconstruction methods of gene tree reconstruction (NJ, ML, and BI) controlled the false positive rates approximately at the desired *α* -level for all choices of *α*-levels (Figure 5C),D. In other words, for each reconstruction method, the plot of false positives (*Y *-axis) versus *α* -levels (*X*-axis) was below the line *y* = *x*.

**Figure 6:**
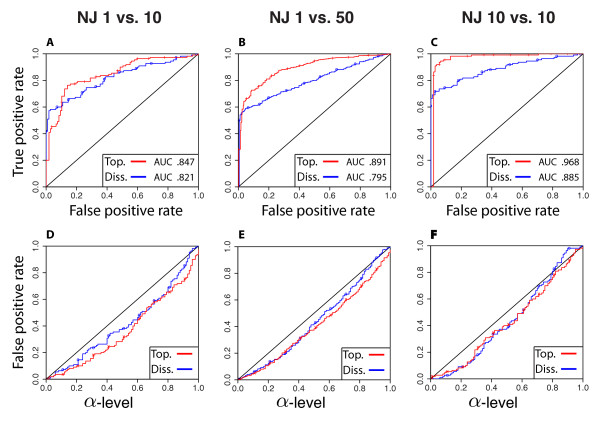
**Graphs depicting the performance of the SVM-based test in detecting differences between gene trees reconstructed from simulated data using NJ.** Trees were reconstructed using PHYLIP. Three different tree comparisons are used: one gene tree from species 1 versus 10 gene trees from species 2 (**A** and **D**), one gene tree from species 1 versus 50 gene trees from species 2 (**B** and **E**), and 10 gene trees from species 1 versus 10 gene trees from species 2 (**C** and **F**). In all graphs, we denote red lines with crosses topological dissimilarity maps and blue lines with circles standard dissimilarity maps of trees. **A**, **B**, and **C** show ROC curves on the simulated data from gene trees generated under different species trees. See the section *ROC Curves and False positive plots* for a description of the ROC curve. **D**, **E**, and **F** show curves for false positive rates vs. *α*-levels on the simulated data with gene trees generated under the same species trees. In **D**, **E**, and **F**, the *X* axis represents the *α*-levels and the *Y * axis represents the false positive rates.

In the evaluation of the performance of our statistical test across different alignment sampling strategies (1 vs. 10; 1 vs. 50; 10 vs. 10), the ROC curves were well above the diagonal and produced larger empirical AUCs (*AUC* ≥ 0.79 ) (Figure 6A)–C, again indicating that our statistical test produced accurate results. Three additional patterns emerged from these results that were worth noting. First, for both types of dissimilarity maps, empirical AUCs were smaller in tests involving single gene alignments (i.e. 1 vs. 10 and 1 vs. 50) (Figure 6A),B compared with tests involving a balanced sampling design (10 vs. 10) (Figure 6C). Second, topological dissimilarity maps resulted in larger empirical AUCs compared with dissimilarity maps (Figures 6A)–C. This pattern was consistent across all three sampling strategies; however, the AUC differences were smallest for 1 vs. 10 and largest for 10 vs. 10. The largest AUC (0.968) was achieved when using the topological dissimilarity map and the 10 vs. 10 sampling strategy (Figure 6c). Third, our results indicated that, under all explored gene alignment sampling strategies, false positive rates were always controlled at the desired *α* -level for all choice of *α*-levels (Figures 6d)–F.

### Computation Time

The running of GeneOut on the eight-taxon data sets required relatively little computation time. The average run time for tests performed with NJ method under a range of gene sampling scenarios ranged from 35.34 to 41.97 minutes. Use of BI required more time, with an average of 2.23 to 2.24 hours. Use of a ML method required substantially greater amounts of computation time, with average of 16.59 to 16.79 hours. These latter two reconstruction methods were only used in tests that involved a 1 vs. 10 sampling strategy.

The running of GeneOut on data sets featuring a larger number of taxa required greater computational time. The average run time of GeneOut for 30-taxa trees using NJ and a 10 vs. 10 sampling scenario required either 8.74 or 16.82 hours using a training/testing set of 100/200 or 200/400 trees, respectively. Correspondingly, increasing the number of taxa to 50 resulted in increased run times of 20.09 and 38.43 hours, and increasing the number of taxa to 75 resulted in computation times of 38.26 and 75.36 hours. As expected, in all analyses that explored the application of GeneOut to trees with greater taxon sampling the estimated p-values were all very small (*p* < 0.01 ), due to the choice of large species tree depths.

## Conclusions

Easier access to the genome now provides the opportunity to collect genetic data, either intentionally or unintentionally, from loci that reflect different underlying evolutionary processes. Analysis of trees in multidimensional space has been used previously as a statistical test of trees in a multi-dimensional vector space; however, this has largely been performed as a test for congruence between two given trees [7,13], and the analysis of large sets of trees in a tree space has been primarily performed as a visualization method, without a corresponding statistical test [6]. Our work here presented a novel statistical hypothesis test for use on multiple sets of trees in a multi-dimensional vector space using SVMs. These results indicated that our SVM-based statistical test is an effective and accurate non-parametric method for statistically discerning between trees that have significantly different distributions in a multi-dimensional space.

Our use of gene trees simulated across a range of species-tree depths provided us with an opportunity to evaluate the performance of our statistical test across a range of multidimensional tree distributions, from those that were virtually indistinguishable from each other (e.g. at species tree depths of 0.1 *N*_*e*_; Additional file 1: Figure S1.) to non-overlapping tree distributions (e.g. depths of at least 4.0*N*_*e*_ ; Additional file 1: Figure S1.). In tests utilizing simulated gene trees (i.e. without gene tree reconstruction) our statistical test appeared to be particularly sensitive in detecting small differences between tree distributions and correctly rejected the null hypothesis for two different sets of gene trees simulated at species tree depths as low as 0.2*N*_*e*_ . This result at this species tree depth was particularly surprising due to the exceptional amount of visually-perceived overlap between tree distributions in PCA ordination space (presumably as a function of substantial incomplete lineage sorting). This accuracy at low species tree depths may be be due to the fact of large sample sizes (10,000 vs. 10,000 ). Such large sample sizes are unlikely to be used in empirical tests where smaller numbers of genes are compared and where tree reconstruction will be employed. However, even when these conditions were factored in to the performance of our statistical test, the ROC and AUC results indicated that it is a robust method for detecting differences between tree distributions. Equally important in the discussion of our statistical tests’ performance is its controlling of false positive rates. In our testing sets of gene trees within the same species tree, our statistical test consistently did not reject the null hypothesis. This was evident in high p-values in the application of our statistical test directly to simulated gene trees (Figure 4), and in ROC curves that were plotted below the diagonal (Figures 5, 6). Both patterns strongly indicate that the power of our statistical test does not come at the expense of a higher probability for false positive rates.

From our simulation study it seems that our statistical test has more power with topological dissimilarity maps than with dissimilarity maps. Ané discussed in [32] that events that changed the tree topology seem more important to detect than events that only modified the tree’s branch lengths. Thus we want to weight more on topological difference between trees than difference on branch lengths. Using topological dissimilarity maps puts most weights on topological difference between trees than difference on branch lengths. This seems to cause our statistical test higher power with topological dissimilarity maps than with the dissimilarity maps.

The generality of our statistical test and its implementation provides a number of benefits. First, the core of our statistical test is based on a non-parametric test, which provides a relatively fast method of analysis. Even when using model-based BI reconstruction methods the majority of our tests required only a couple hours of computation time. Expanded taxon sampling to as many as 75 taxa pushed computation times into the 1–3 day range, which we see as very acceptable computation time in the current field of model-based multi-locus phylogenetics. Second, our statistical tests’ use of reconstructed tree distributions through bootstrapping or sampling from a posterior distribution is expected to help mitigate the problem of tree reconstruction error. This is a likely contributor to the low probability of false positives seen in the ROC plots. Additional file 1: Figure S3. This may also explain the lack of substantial differences in results based on NJ, ML, and BI reconstruction methods: even though one method may provide a more consistent point estimate of a tree, they may all generate similar tree distributions. Third, our statistical test has the flexibility to compare tree distributions for a range of combination of genes. This accommodates tests confirming outlier gene tree behavior for a single gene relative to a larger collection of genes sampled from the same taxonomic group, but could also accommodates the comparison of two multi-gene sets. In fact, our 10 vs. 10 tests with GeneOut demonstrated an improved performance over those involving a single gene (i.e. 1 vs. 10 or 1 vs. 50). This is perhaps due to the fact that a statistical test with two independent samples works well with balanced samples, because the variances of the two samples are approximately equal under the null [33]. In any case, the multi-gene version of our statistical test may be particularly useful in the comparison of gene trees from putative host-parasite taxa to test for co-evolution. Finally, while we used dissimilarity and topological dissimilarity maps to define the vector space of trees, our statistical test can be applied to vector spaces derived from a wide range of tree metrics, such as Robinson-Foulds distances [34] and quartet distances [35].

Systematists often aim to statistically evaluate competing phylogenetic hypotheses with a single gene or concatenated set of genes by comparing trees reconstructed with and without a topological constraint [1,36]. Our statistical test can serve as a novel approach for testing the distributions of trees that result from these comparisons. Multi-dimensional visualization of trees sampled from independent Bayesian phylogenetic analyses has been proposed as a method for assessing convergence of Markov chains on the posterior distribution [6] and our statistical test can add a statistical edge to this approach. Finally, as noted above, our statistical test may be useful for testing hypotheses of coevolution (e.g. in host/parasite systems) by testing sets of genes from each of the potentially coevolving groups. This is not meant to be an exhaustive list of applications, and we envision that our statistical test and the SVM-based test that it is based on can be applicable to any situation where there is the potential to compare two distributions of trees. Note that this method is not meant to be used for detecting outliers from a set of trees. If we apply this method for the post-hoc analysis for detecting outliers we have to conduct multiple comparisons and this causes higher false positive rates. Thus if one wants to apply this method for detecting outliers, a correction for multiple comparisons, such as Bonferroni correction, should be applied.

While the non-parametric nature of our statistical test has the upside that it can be applied to tests of discordance between two sets of trees caused by a range of reasons, the flip-side is that it does not provide an ability to draw specific conclusions about the underlying cause for significant differences between tree distributions. Subsequent model-based analyses that can identify specific genetic processes (e.g. selection [37] or recombination [38]) can then be used to identify the potential underlying causes. We also note that the supervised nature of the SVM algorithm will limit the exhaustive application of our statistical test to data sets containing large numbers of genes, and that for these situations, some basic information must be provided regarding the potential comparisons to be made. There have been several attempts to cluster trees in a multi-dimensional framework [39,40], and it is possible that unsupervised learning techniques, such as *k*-means clustering or quality threshold (QT) clustering, can serve as an important addition to our SVM-based method by identifying hypothetical sets of trees to be tested.

### Software

The software GeneOut is freely available at http://cophylogeny.net/SVM.php. The core of the software was written in C++ and unix shell scripting. GeneOut reads in alignments and parameters specified in Nexus format [41].

## Appendix

### GeneOut Algorithm

**Input:** Two sets of alignments, *A* and *B*, sample size *M* for training phase and *N* for testing phase.**Output:** p-value under the null hypothesis that the trees underlying *A* and *B* are drawn from the same distribution.

Set *m*_*A*_ := *ceil*(*M*/|*A*|) and *m*_*B*_ := *ceil*(*M*/|*B*|).

 For each alignment in *A*, reconstruct *m*_*A*_ trees.

 For each alignment in *B*, reconstruct *m*_*B*_ trees.

 Let *V*_*A*_ := set of trees generated from *A*.

 Let *V*_*B*_ := set of trees generated from *B*.

 Train SVM on data (*V*_*A*_,*V*_*B*_).

 Set *n*_*A*_ := *ceil*(*N*/|*A*|) and *n*_*B*_ := *ceil*(*N*/|*B*|).

 For each alignment in *A*, reconstruct *n*_*A*_ trees.

 For each alignment in *B*, reconstruct *n*_*B*_ trees.

 Let *R*_*A*_ :=  set of trees generated from *A*.

 Let *R*_*B*_ := set of trees generated from *B*.

 Let *δ*_0_ := Separation percentage between *R*_*A*_ and *R*_*B*_.

 Set count := 0.

**for***i* = 1,…,*k***do**

 Order the alignment sets arbitrarily, *A* = 

(*a*_1_,…,*a*_*ℓ*_),*B* = (*b*_1_,…,*b*_*m*_).

 Randomly permute set membership labels of

 alignments in *A,B* to obtain *A*^*′*^,*B*^*′*^.

 For each $a_{i}^{\prime }$, replace with a bootstrap of |*a*_*i*_| columns

 of $a_{i}^{\prime }$.

 For each $b_{i}^{\prime }$, replace with a bootstrap of |*b*_*i*_| columns

 of $b_{i}^{\prime }$.

 For each alignment in *A*^*′*^, reconstruct *m*_*A*_ trees

 For each alignment in *B*^*′*^, reconstruct *m*_*B*_ trees.

 Let $V_{A}^{\prime }:=$ set of trees generated from *A*^*′*^.

 Let $V_{B}^{\prime }:=$ set of trees generated from *B*^*′*^.

 Train SVM on data $\left(V_{A}^{\prime },V_{B}^{\prime }\right)$

 For each alignment in *A*^*′*^, reconstruct *n*_*A*_trees.

 For each alignment in *B*^*′*^, reconstruct *n*_*B*_ trees.

 Let $R_{A}^{\prime }:=$ set of trees generated from *A*^*′*^.

 Let $R_{B}^{\prime }:=$ set of trees generated from *B*^*′*^.

 Let *δ*:= Separation percentage between $R_{A}^{\prime }$ and $R_{B}^{\prime }$.

**if***δ* ≤ *δ*_0_**then**

 count := count + 1.

end if

end for

 Output p-value := count / k.

## Competing interests

The authors declare that they have no competing interests.

## Authors contributions

DH developed methods and algorithms, wrote all software and testing scripts, generated simulation data, ran all simulations, and drafted and revised the manuscript. PH developed methods and algorithms, and drafted and revised the manuscript. EO designed simulation, and drafted and revised the manuscript. DW supervised and coordinated this project, designed simulation, analyzed the simulation results, and drafted and revised the manuscript. RY developed methods and algorithms, designed statistical analysis on the simulation results, supervised and coordinated this project, analyzed the simulation results, and drafted and revised the manuscript. All authors read and approved the final manuscript.

## Authors’ information

Join first authors: David C. Haws and Peter Huggins. Joint last authors: David W. Weisrock and Ruriko Yoshida.

## Supplementary Material

Additional file 1 **MrBayes****parameters.** All Bayesian analyses were run using MrBayes. Two independent runs were performed for each data set, each using four Markov chains and the default temperature parameter setting of 0.2. 100,000 generations were run with a sample drawn every 100 generations and 25% of the samples treated as burn-in. The minimum, first quartile, median, second quartile, and maximum of all 2,640,000 split frequencies (observed across all simulations) were 0.0, 0.003497, 0.007667, 0.010443, 0.098460. **Figure S1**. Fifteen data sets, with 100 gene trees (blue diamonds) generated under a coalescent model under a species tree S1, and 100 gene trees (red circles) generated via coalescence under a different species tree S2. All fifteen data sets had a fixed effective population size of 1 Ne individuals. The first two PCA components were used to plot gene trees in two-dimensional space. PCA projections were computed using R [31]. **Figure S2**. Fishers linear discriminant for 20,000 gene trees generated under either the same species tree (blue) or two different species trees (red). Gene trees were vectorized using the dissimilarity map. The dashed line at FDL = 1 indicates where the variance between gene trees is equal to the variance within gene trees. Values of FLD that are greater than 1 suggests clear separation between sets of gene trees. **Figure S3**. Graphs depicting the performance of the SVM-based test in detecting differences between gene trees reconstructed from simulated data using NJ, BI, and ML. Trees were reconstructed using PHYLIP, MrBayes and PhyML. One gene tree from species 1 vs. 10 gene trees from species 2. In all graphs, both topological dissimilarity maps (red crosses) and standard dissimilarity maps (blue circles) of trees are considered. Top panels: ROC curves on the simulated data where gene trees are taken from different species trees. See the section Simulation Study of GeneOut for a description of the ROC curve. Bottom: false positive rates were plotted where gene trees are taken from the same species trees. The X-axis is the ?-level and the Y-axis gives the corresponding false positive rate. Click here for file
