# Supplementary material for: A support vector machine based test for incongruence between sets of trees in tree space
Source: BMC Bioinformatics. 2012 Aug 21;13:210. doi: 10.1186/1471-2105-13-210 (PMC3536566; doi:10.1186/1471-2105-13-210)
Supplement: Additional file 1 — MrBayesparameters. All Bayesian analyses were run using MrBayes. Two independent runs were performed for each data set, each using four Markov chains and the default temperature parameter setting of 0.2. 100,000 generations were run with a sample drawn every 100 generations and 25% of the samples treated as burn-in. The minimum, first quartile, median, second quartile, and maximum of all 2,640,000 split frequencies (observed across all simulations) were 0.0, 0.003497, 0.007667, 0.010443, 0.098460. Figure S1. Fifteen data sets, with 100 gene trees (blue diamonds) generated under a coalescent model under a species tree S1, and 100 gene trees (red circles) generated via coalescence under a different species tree S2. All fifteen data sets had a fixed effective population size of 1 Ne individuals. The first two PCA components were used to plot gene trees in two-dimensional space. PCA projections were computed using R [31]. Figure S2. Fishers linear discriminant for 20,000 gene trees generated under either the same species tree (blue) or two different species trees (red). Gene trees were vectorized using the dissimilarity map. The dashed line at FDL = 1 indicates where the variance between gene trees is equal to the variance within gene trees. Values of FLD that are greater than 1 suggests clear separation between sets of gene trees. Figure S3. Graphs depicting the performance of the SVM-based test in detecting differences between gene trees reconstructed from simulated data using NJ, BI, and ML. Trees were reconstructed using PHYLIP, MrBayes and PhyML. One gene tree from species 1 vs. 10 gene trees from species 2. In all graphs, both topological dissimilarity maps (red crosses) and standard dissimilarity maps (blue circles) of trees are considered. Top panels: ROC curves on the simulated data where gene trees are taken from different species trees. See the section Simulation Study of GeneOut for a description of the ROC curve. Bottom: false positive rates were plotted wh [file 1471-2105-13-210-S1.pdf]

Supplement

`MrBayes` parameters

All Bayesian analyses were run using `MrBayes`. Two independent runs were performed for each data set, each using four Markov chains and the default temperature parameter setting of 0.2. $100,000$ generations were run with a sample drawn every 100 generations and 25% of the samples treated as burn-in. The minimum, first quartile, median, second quartile, and maximum of all 2,640,000 split frequencies (observed across all simulations) were 0.0, 0.003497, 0.007667, 0.010443, 0.098460.

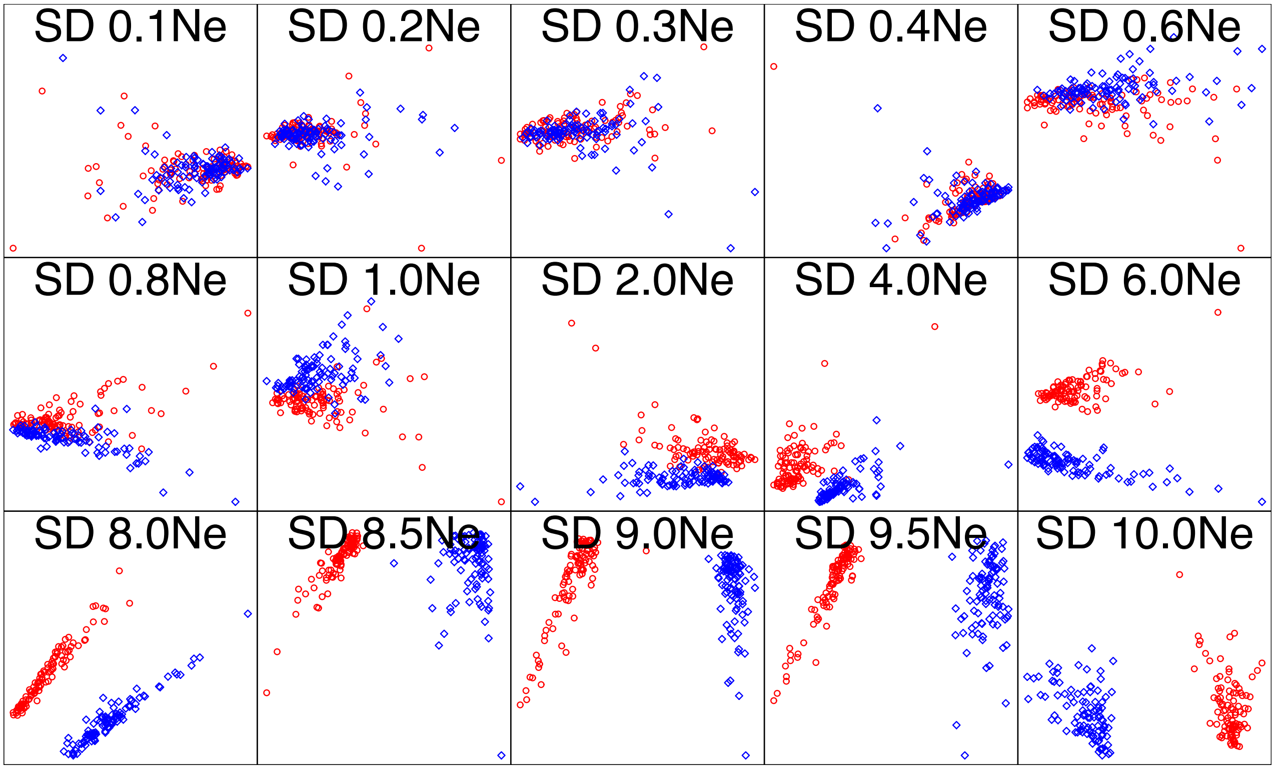

Figure 1: Fifteen data sets, with 100 gene trees (blue diamonds) generated under a coalescent model under a species tree S1, and 100 gene trees (red circles) generated via coalescence under a different species tree S2. All fifteen data sets had a fixed effective population size of 1 N_e individuals. The first two PCA components were used to plot gene trees in two-dimensional space. PCA projections were computed using R [31].

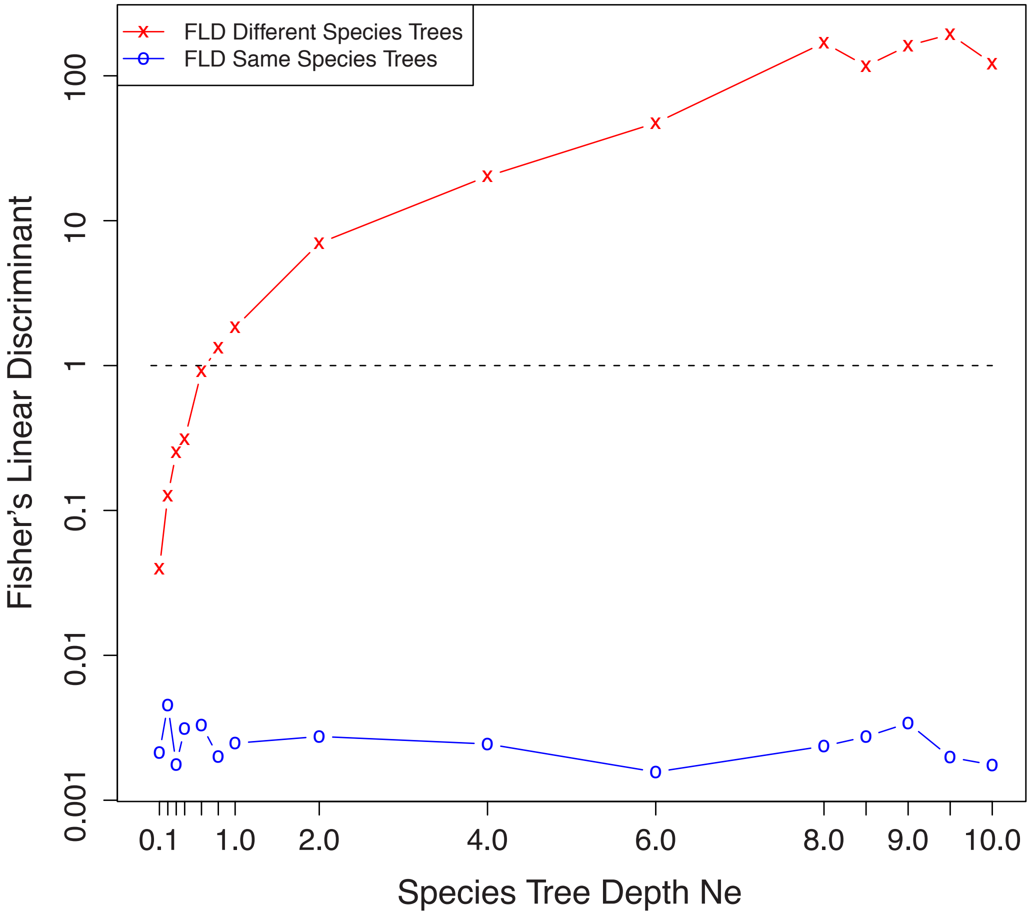

Figure 2: Fisher's linear discriminant for 20,000 gene trees generated under either the same species tree (blue) or two different species trees (red). Gene trees were vectorized using the dissimilarity map. The dashed line at FDL = 1 indicates where the variance between gene trees is equal to the variance within gene trees. Values of FLD that are greater than 1 suggests clear separation between sets of gene trees.

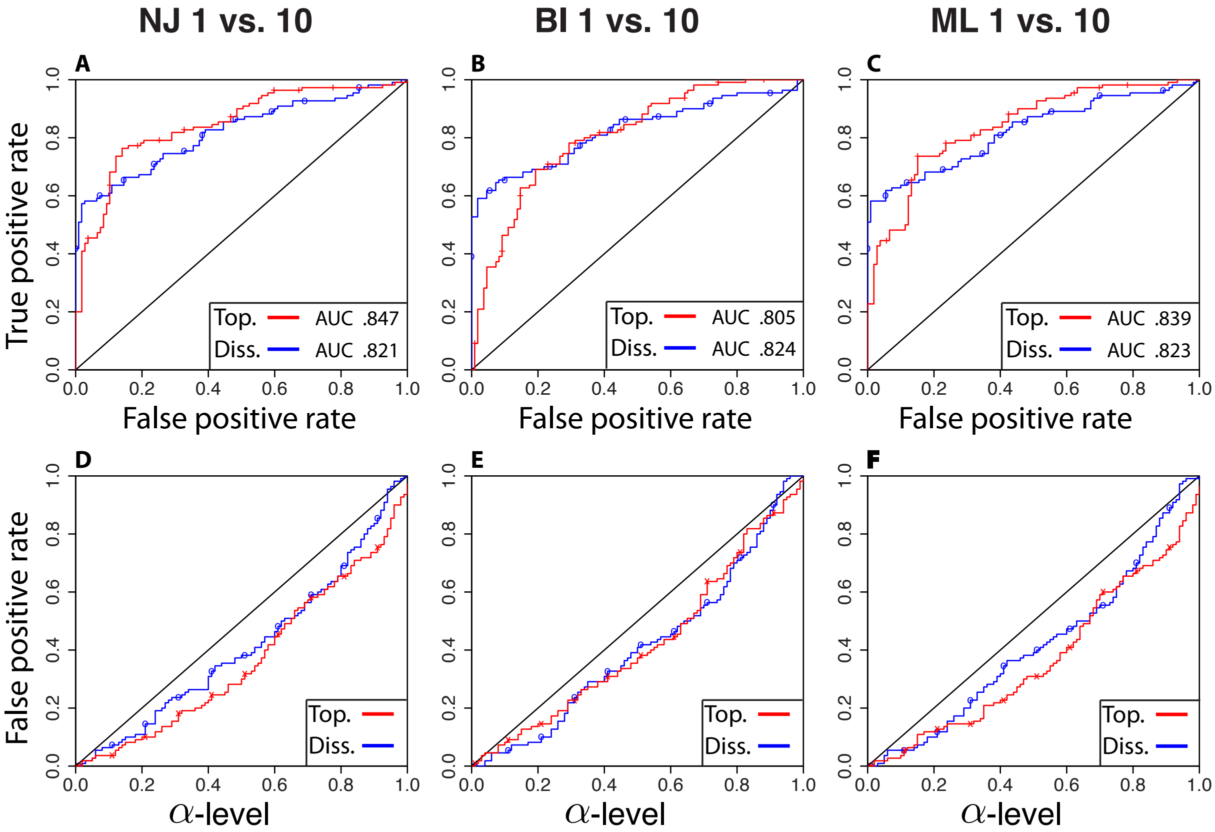

Figure 3: Graphs depicting the performance of the SVM-based test in detecting differences between gene trees reconstructed from simulated data using NJ, BI, and ML. Trees were reconstructed using `PHYLIP`, `MrBayes` and `PhyML`. One gene tree from species 1 vs. 10 gene trees from species 2. In all graphs, both topological dissimilarity maps (red crosses) and standard dissimilarity maps (blue circles) of trees are considered. Top panels: ROC curves on the simulated data where gene trees are taken from different species trees. See the section Simulation Study of `GeneOut` for a description of the ROC curve. Bottom: false positive rates were plotted where gene trees are taken from the same species trees. The X-axis is the α-level and the Y-axis gives the corresponding false positive rate.
